# Are migraineur women really more vulnerable to stress and less able to cope?

**DOI:** 10.1186/1472-6963-8-211

**Published:** 2008-10-10

**Authors:** Mintaze Kerem Gunel, Fatma Yildirim Akkaya

**Affiliations:** 1 Hacettepe University, Faculty of Health Sciences, Department of Physical Therapy and Rehabilitation, 06100, Ankara, Turkey; 2 Ankara University, Faculty of Political Science, Department of Labor Economics and Industrial Relations, Ankara, Turkey

## Abstract

**Background:**

In this study, we aimed to investigate the differences between a sample of migraineurs and non-migraineurs with regard to their stress symptoms, tendency to stress, coping styles and life satisfaction.

**Methods:**

This study was carried out on a migraineur group (n = 62, mean age: 37.5 ± 11.3, range: 18 to 61 years) and a non-migraineur group (n = 58, mean age: 32.0 ± 11.2, range: 18 to 61 years). Stress Audit (Symptoms), Stress Audit (Vulnerability), Turkish version of Ways of Coping Inventory Scales and Life Satisfaction were applied to the migraineur and non-migraineur groups.

**Results:**

No significant differences were found between the groups in the scores of the stress symptoms except in the sub scores of the sympathetic system. There was no significant difference between the groups in the tendency to stress and life satisfaction (p > .05). For scores of the coping styles, the mean scores of the seeking social support subscale was higher in the control group than that of the migraineur group. However, migraineur women had higher mean scores in the submissive and the optimistic subscales.

**Conclusion:**

We consider that, these outcomes may emphasize the necessity to be careful when using negative expressions about stress relating to migraineurs. Further comprehensive studies are required considering the multiple triggers of the disease in various cultural contexts.

## Background

Migraine is one of the most common public health problems with high psychosocial and economical burden [1,2]. It is a disease with multifactorial origin, and migraine headache can be triggered by endogeneous (e.g. stress, insomnia) and exogenous (e.g. food, nitroglycerine) stimuli [3,4]. Among these stimuli, stress is frequently considered to precipitate, exacerbate, and maintain migraine [5]. Stress induces autonomic arousal, particularly sympathetic system activation, which is also involved in the neurovascular psychopathology of migraine attacks [1,6]. In general, stress is a specific adaptive reaction of an organism to a variety of physical or psychological challenges. This specific stress reaction depends on the nature and intensity of the stressor, on the social and cultural context, and on the subject's ability to evaluate and to cope with the problem as well as his vulnerability to stress [1,7]. Stress has repeatedly been shown to trigger acute migraine attacks. Therefore, recent studies focus on specific stressful situations and coping strategies in patients with migraine [8,9].

It is suggested by authors that there is a preexistent psychological vulnerability induced by stress which keeps migraineurs in constant adaptive efforts, thus maintaining stress. So, stress influences migraine negatively, and recurrent migraine attacks can produce stress in the end [4]. The coping style is another mediating factor in the relationship between stress and migraine. Coping ways can be either functional, such as seeking social support, optimistic styles, or dysfunctional styles such as submissive ways of coping [10]. It is posited that the ways to cope with stress can be an important determinant of stress-related symptoms that negatively affect an individual's health and social functioning [11].

The theory of stress proposed by Lazarus and Folkman posits that an individual's cognitive appraisal of an event can affect both the stress response and the coping efforts of that individual [6]. Some studies about migraineurs indicated migraine to be associated with more life stressors which are often appraised as threatening and coped in more ineffective ways and indicated that migraineurs assess stressful life events worse and use less effective coping styles compared with the non-migraineurs [11-14]. These findings indicated that migraineurs may exacerbate the impact of their headache pain by perceiving stressful events more negatively than non-migraineurs [15]. Based on clinical observations, migraine patients were described as ambitious, perfectionist, rigid, obsessional, and very achievement oriented people [12,13]. However, some other studies showed that migraineurs did not have difference in the tendency to stress compared with non-migraineurs [16-18].

Migraine is much more common in women than in men. The prevalence rates were found to be 21.8% in women and 10.9% in men (16.4% in the general population aged between 15 and 55 years) in Turkey, similar to those in the literature [19]. Thus, in the present study the sample consisted of women.

Although there is not a definite consensus, suggestions on a higher tendency to stress, less effective coping styles with stress, and low life satisfaction in migraineurs predominate in the literature [14,20,21]. It was aimed to investigate further the differences between migraineurs and non-migraineurs on account of stress symptoms, tendency to stress, coping styles and life satisfaction.

## Methods

### Participants

All volunteer subjects were asked to give their written informed consent together with the completed questionnaire. The study was approved by the Hacettepe University Ethical Committee, number of HEK 08/157. Inclusion criteria were being over 18 years of age and being literate.

#### Migraineurs group

Clinicians (general practitioners, neurologists) from various health care facilities (primary health care centers, neurology clinics at hospitals) were informed about the aim of the study and asked to refer their women diagnosed with migraine according to the IHS (International Health Society) diagnostic criteria [22], excluding subjects who had a chronic physical disorder.

Initially a total of 100 migraineurs were recruited, and 72 of the questionnaires were returned. Properly and totally filled 62 (mean age: 37.56 ± 11.32 (range: 18 to 61 years) questionnaire forms were taken in the analysis. We had no information about headache history duration, no information about co morbid tension-type headache and no information about how many patients had migraine with aura.

#### Control group

The control group was formed of non-migraineurs by using the snowball sampling method on voluntary basis. A total of two hundred women in the control group who were in the similar age range with the migraineurs group filled in the forms. The filled forms were returned by the participants in closed and stamped envelopes to be sent back to the researchers. A total of 157 control subjects returned the questionnaire forms of which 143 were properly filled. Fifty-eight non-migraineur subjects who had similar age, marital, educational, occupational and economic status as that of the patients were selected out of 143 control subjects (mean age: 32.07 ± 11.26, range: 18 to 61 years). No differences were found between the groups in any of the socio-demographic variables except for age (Table 1).

**Table 1:** Distribution of socio-demographic characteristics in migraineurs and non-migraineurs

	**Migraineurs**		**Non-migraineurs**	
	**Min.**	**Max.**	**Min.**	**Max.**
	
Age Range (year)**	**N**	**%**	**N**	**%**

18–32	25	40.3	37	60.3
33–47	20	32.3	17	29.3
48–61	16	25.8	6	10.3
Educational Status*				

Primary	11	17.7	4	6.9
Secondary	14	22.6	12	20.7
High	28	45.2	28	48.3
Post-graduate	9	14.5	14	24.1
Economic Status*				

Low	13	21	12	20.7
Moderate	40	64.5	37	63.8
High	9	14.5	9	15.5
Marital Status*				

Single	16	25.8	22	37.9
Married	44	71	32	55.2
Divorced	2	3.2	4	6.9
Occupational Status*				

Work	38	61.3	32	55.2
Non-working	24	38.7	26	44.8

Min: Minimum, Max: Maximum, SD: Standard Deviation, *Chi-Square test p > .05, ** Chi-Square test p < .05

The socio-demographic characteristics of the migraineurs and non-migraineurs are given in Table 1.

### Instruments

For assessing stress symptoms and tendency to stress, the two subscales of the adapted version of the Stress Audit in Turkish by Sahin and Durak was used [10,22-24].

The other assessment instrument was the Coping Styles Inventory (CSI) developed by Sahin and Durak based on the Ways of Coping Inventory [25,26]. The seven stress symptoms subscales of the Stress Audit 4.2-OS were used in this study: the problems of the muscle system, parasympathetic system, sympathetic system, emotional system, cognitive system, endocrine system and immune system. This part of the instrument consists of 70 items and each subscale consists of 10 items.

The tendency to stress subscale of the Stress Audit 4.2-OS consists of 20 items.

The Coping Styles Inventory consisting of 30 items was additionally applied to the subjects. This inventory has 5 subscales: self-confident (7 items), optimistic (5 items), helpless (8 items), submissive (6 items) and seeking social support (4 items).

Life satisfaction was assessed on a 7-point Likert scale using a global one-item measure ("I am satisfied with my life") with alternatives ranging from (1) least satisfied, to (7) very much satisfied.

### Data Analysis

Data analyses were performed using the SPSS version 13.0 (SPSS Inc., Chicago, IL, USA). A significance level of P < 0.05 was chosen, and all tests were two-sided. Results were analyzed by using the t-test and χ^2 ^test. The correlation between age and stress symptoms, coping styles, tendency to stress, total scores and life satisfaction in both groups was calculated using the Pearson correlation method.

Relative effect size was calculated using the statistical data obtained from the study.

Relative Effect Size = [(mean migraineurs - mean non-migraineurs)/pooled SD]. Pooled SD was calculated using the following formula;

$$\text{Pooled SD}=\surd \frac{{\text{SD}}_{1}^{2}({\text{n}}_{1}-1)+{\text{SD}}_{2}^{2}({\text{n}}_{2}-1)}{\text{n}1+\text{n}2-2}$$

The results were assigned as 0.2 indicative of a small effect, 0.5 a medium and 0.8 a large effect size [27].

The power to detect a small, medium and large effect in the two-sample Student's t-test with 62 and 58 subjects is 19%, 78% and 99% respectively [28].

## Results

When stress symptoms scores of each subscale were compared, no difference was found between the migraineur group and control group, except in the sympathetic system subscale (t = 3.58, p < .05). In addition, no significant differences were found between the groups in tendency to stress and life satisfaction scores (p > .05).

For coping styles scores, seeking social support subscale mean score in the control group was higher than that of the migraineur group (t = -3.23, p < .05), whereas the migraineur group had higher mean scores in the submissive (t = 2.44, p < .05) and optimistic subscales (t = 2.11, p < .05), (Table 2). The results of relative effect size relating to stress symptoms, coping styles, tendency to stress, total scores and life satisfaction are given in Table 2.

**Table 2:** Comparison of Stress Symptoms, Tendency to Stress, Coping Styles and Life Satisfaction between Migraineurs and Non-migraineurs

**Stress Symptoms**	**Migraineurs n:62**		**Non-migraneurs n:58**		**Student t test**		**Relative effect size**
	
	**Mean**	**SD**	**Mean**	**SD**	**t**	**P**	
Muscle system	22.6	11.1	21.9	16.2	0.27	0.79	0.05
Parasympathetic system	20.4	13.6	19.4	10.4	0.46	0.65	0.08
Sympathetic system	21.7	12.7	14.1	10.5	3.58*	**0.00**	0.65
Emotional system	29.0	21.1	28.1	15.7	0.26	0.79	0.05
Cognitive system	23.6	13.5	25.5	12.4	0.27	0.41	0.15
Endocrine system	14.0	10.0	15.4	8.2	-0.8	0.43	0.15
Immune system	14.8	10.7	13.3	10.7	0.77	0.45	0.14

**Coping styles**							

Seeking social support	8.2	3.7	10.4	3.7	-3.23*	**0.00**	0.59
Submissive	6.1	3.7	4.7	2.6	2.44*	**0.02**	0.45
Self-confident	13.5	4.6	12.3	4.2	1.48	0.14	0.27
Optimistic	9.2	5.5	7.5	3.1	2.11*	**0.04**	0.37
Helpless	10.4	4.9	10.4	4.8	-0.06	0.95	0.01

**Total scores of Tendency to stress**	46.7	10.0	48.4	12.7	-0.81	0.42	0.15

**Life Satisfaction**	4.83	1.79	4.94	1.59	0.73	0.73	0.06

SD: Standard Deviation, * p < .05

In the correlation analyses between age and stress symptoms, coping styles, tendency to stress, total scores and life satisfaction in both groups, age in migraineurs was found to be correlated only with the immune system among the stress symptoms (r = 0.45, p < .01), and in non-migraineurs age was found to be correlated with the endocrine system among the stress symptoms (r = -0.29, p < .05).

## Discussion

According to our results, the fact that there was no difference between migraineurs and non-migraineurs with regard to stress symptoms (except for sympathetic system), tendency to stress and life satisfaction, self-confident and helpless subscales of coping styles, while there was difference against migraineurs in seeking social support subscale of coping styles, and difference in favor of migraineurs in submissive and optimistic subscales, have led us to believe that our results may emphasize the possibility of different states relating to stress in migraineurs.

In the light of the literature, migraineurs were expected to have higher stress symptoms and tendency to stress, but lower life satisfaction, and to use non-effective coping styles compared with the non-migraineurs [1,12,13,16,17].

Looking at our results more closely with regard to stress symptoms reveals that there is no significant difference between migraineurs and non-migraineurs except the sympathetic system symptoms which were found more commonly among the migraineurs. The clinical symptoms of migraine are widely accepted to be related to the involvement of the autonomic nervous system, and especially to dysfunction in the regulation of the circulatory system and autonomic balance [4,29]. Siniatchkin et al. studied the neurophysiological reactivity to stress in migraineurs and compared their results with those of healthy subjects and reported that migraine patients were characterized by increased neurophysiological and autonomic reactivity to stress [30]. The sympathetic nervous system (SNS) plays a role in migraine pathophysiology. Migraineurs, compared to non-migraineurs, have a relative deficit in SNS function during the baseline or interictal phase of headaches [31]. Sympathetic symptoms are not necessarily always psychological, but may have some constitutional basis [32].

Some researchers reported that prediction of migraine was dependent not just on stress but also on the person's appraisal of events as threatening, and whether resources were available to cope with the stress. They suggested that the simple examination of stress is insufficient, and it is necessary to understand the emotional processes associated with stressful events, as well as the person's responses to these emotions [13,15]. Thus, coping with stress should also be taken as an intrinsic process in response to stress. Therefore, ways of coping with regard to migraine were also considered in this study.

In the present study, it was found that migraineur patients used the coping way of seeking social support less compared to the control group individuals.

Holm et al. stated that "Tension headache sufferers may use coping strategies such as internalizing more often and seeking social support less often than adults without headache" [33], which is in accord with this result of our study.

This may be because of their fear of the experience of pain which leads to avoiding stimuli that they might trigger pain, including social interactions and situations [15]. Thus, not seeking social support by the migraineurs may be the result of migraine in stead of the cause of migraine headache.

Our results indicate that migraineurs are more submissive and optimistic in coping with problems compared with non-migraineurs. It was previously suggested that migraineurs use less effective coping styles and this suggestion almost became a general judgment in the literature [14]. On the other hand, other studies indicated that coping styles did not differ between migraineurs and non-migraineurs [5]. Our findings are further supported with those of some previous studies [21,34]. One explanation for the mentioned inconsistency of study results may be variability in methodology [17].

The results of the present study indicate that migraineur women do not differ from non-migraineur women in tendency to stress and stress symptoms except sympathetic symptoms, and life satisfaction; however they differ from non-migraineur women in coping styles including seeking social support, submissive and optimistic styles. Thus, it cannot be suggested that migraineurs always use less effective coping styles compared with the non-migraineurs, as indicated by our findings, and having migraine is not the only determinant of the reaction to stress.

Many other psychosocial factors would be effective in determining the ways of coping with stress. Still, intervention in ineffective coping styles would be a significant component of the whole psychosocial management of migraine besides medical treatment. In a recent study, it was found that this variable was a key predictor of response to treatment and it was individuals who utilized social support before treatment who benefited most from treatment, and it was individuals who increased migraineurs use of social support across the treatment period who benefited most [35].

There were several limitations to the study. One of these limitations was that we had no information about headache history duration, no information about co morbid tension-type headache and no information about how many patients had migraine with aura. Another important limitation was that we were not able to do a paired match for age. While the mean age of migraineurs was 37.56 ± 11.32 years, the mean age of non-migraineurs was 32.07 ± 11.26 years. Considering that age could affect stress symptoms, tendency to stress, coping styles and life satisfaction, we examined the correlation between age and these parameters and found no correlation. Although not finding correlation between age and stress symptoms, tendency to stress, coping styles and life satisfaction was a positive outcome, being able to do a statistically paired match for age as we could for marital, educational, occupational and economic status may have strengthened our study. We would also like to report that the power of the study is moderate and that small differences will not necessarily be detected in the statistical analysis.

In addition, we would like to point out that when we examined the relative effect sizes among the stress symptoms and coping styles, it was found that most effects were neglible (<0.2), there were small effect sizes between 0.2 and 0.49 in submissive, self-confident and optimistic subscales, medium effect sizes between 0.5 and 0.79 in seeking social support and the sympathetic system, while large effect sizes above 0.8 were not found (Table 2).

This study can be regarded as preliminary. The strong part of this study is that it provides needed information from a culture from where not much information is available in the headache literature so far.

Further studies investigating the relationship between psychosocial factors and various aspects of migraine such as type, duration and frequency of pain on larger samples with more detailed exclusion criteria would be informative. In the present study, coping and tendency to stress were evaluated as state characteristics in migraine. Investigating the relationship of trait characteristics, like personality traits, with migraine would take our findings a step further.

## Conclusion

It can be suggested that migraineurs can not always be expected to have an increased tendency to stress, and less functional coping styles as a generalization, as indicated by the results of the present study. Such a generalization has the risk of stigmatization of migraine patients by both the clinicians and the society. Lack of difference between migraineurs and non-migraineurs in their level of life satisfaction according to our results additionally support this suggestion. Still, intervention with dysfunctional coping styles, and supporting functional ways of coping, such as seeking social support [4,36], would possibly prevent recurrence of migraine attacks. Moreover, migraine is a multifactorial neurophysiological syndrome where psychological factors never solely determine the progress of migraine. Further studies considering the multifactorial nature of migraine done in various cultural contexts would elucidate the psychological aspects of migraine.

## Competing interests

The authors declare that they have no competing interests.

## Authors' contributions

MKG carried out study design, data collection, statistical analysis, data interpretation, manuscript preparation, literature search; FYA carried out study design, data collection, statistical analysis, data interpretation, manuscript preparation, literature search.

## Pre-publication history

The pre-publication history for this paper can be accessed here:


